# Correlating enzyme annotations with a large set of microbial growth temperatures reveals metabolic adaptations to growth at diverse temperatures

**DOI:** 10.1186/s12866-018-1320-7

**Published:** 2018-11-06

**Authors:** Martin K. M. Engqvist

**Affiliations:** Department of Biology and Biological Engineering, Chalmers University of Technology, Gothenburg, Sweden

**Keywords:** Optimal growth temperature, Enzyme functions, Temperature adaptation of metabolism, Archaeal and bacterial metabolism, Protein domains of unknown function

## Abstract

**Background:**

The ambient temperature of all habitats is a key physical property that shapes the biology of microbes inhabiting them. The optimal growth temperature (OGT) of a microbe, is therefore a key piece of data needed to understand evolutionary adaptations manifested in their genome sequence. Unfortunately there is no growth temperature database or easily downloadable dataset encompassing the majority of cultured microorganisms. We are thus limited in interpreting genomic data to identify temperature adaptations in microbes.

**Results:**

In this work I significantly contribute to closing this gap by mining data from major culture collection centres to obtain growth temperature data for a nonredundant set of 21,498 microbes. The dataset (10.5281/zenodo.1175608) contains mainly bacteria and archaea and spans psychrophiles, mesophiles, thermophiles and hyperthermophiles. Using this data a full 43% of all protein entries in the UniProt database can be annotated with the growth temperature of the species from which they originate. I validate the dataset by showing a Pearson correlation of up to 0.89 between growth temperature and mean enzyme optima, a physiological property directly influenced by the growth temperature. Using the temperature dataset I correlate the genomic occurance of enzyme functional annotations with growth temperature. I identify 319 enzyme functions that either increase or decrease in occurrence with temperature. Eight metabolic pathways were statistically enriched for these enzyme functions. Furthermore, I establish a correlation between 33 domains of unknown function (DUFs) with growth temperature in microbes, four of which (DUF438, DUF1524, DUF1957 and DUF3458_C) were significant in both archaea and bacteria.

**Conclusions:**

The growth temperature dataset enables large-scale correlation analysis with enzyme function- and domain-level annotations. Growth-temperature dependent changes in their occurrence highlight potential evolutionary adaptations. A few of the identified changes are previously known, such as the preference for menaquinone biosynthesis through the futalosine pathway in bacteria growing at high temperatures. Others represent important starting points for future studies, such as DUFs where their occurrence change with temperature. The growth temperature dataset should become a valuable community resource and will find additional, important, uses in correlating genomic, transcriptomic, proteomic, metabolomic, phenotypic or taxonomic properties with temperature in future studies.

**Electronic supplementary material:**

The online version of this article (10.1186/s12866-018-1320-7) contains supplementary material, which is available to authorized users.

## Background

The physiology and evolutionary adaptations of living organisms are shaped by the environmental factors that define the habitat in which they live, but availability of metadata describing the environment of each habitat is limited. This is true for such properties as growth temperature, pH, salinity and many more. Several projects addressing aspects of this limitation have been published in recent years. The most comprehensive of these is BacDive [1, 2] which was created by digitizing and mining analog records containing microbial metadata [3]. Other resources include MediaDB, which offers a hand-curated database for microbial growth conditions [4] and KOMODO which supplies media compositions for many thousand organisms [5]. Records regarding the phenotypic and environmental tolerance for over 5,000 species has also been made available [6]. One recent approach leveraged text mining to infer a large number of phenotypic traits from the scientific literature and the World Wide Web [7].

Among environmental factors temperature is unique in that it crosses physical barriers. As a result, organisms cannot efficiently shield themselves from temperature in the way they can shield themselves from extreme external pH or salinity by maintaining steep concentration gradients over biological membranes. Instead, each biomolecule inside microorganisms must be adapted to the temperatures in which they grow. This makes proteins and metabolites from microbes growing at hot temperatures particularly interesting for biotechnological and industrial applications [8]. Organisms can be generally categorized based on the temperature range of their optimal growth. No consensus has been reached regarding the exact temperature range of each category, here I make use of the following: psychrophiles (< 15 °C), mesophiles (15–50 °C), thermophiles (50–80 °C) and hyperthermophiles (> 80 °C).

A range of specific adaptations to high temperatures are known, with many studies focusing on characteristics of thermophilic genomes or molecules such as proteins and lipids [9, 10]. Stability of thermophilic proteins is attributed to their hydrophobic cores, increased numbers of charged residues and disulphide bonds [9, 11, 12]. Cell membranes in thermophiles are characterised by high permeability barrier and capacity to maintain the liquid crystalline phase, due to presence of saturated fatty acids in bacteria, and ether lipids in archaea [13]. Adaptations on the DNA level are also known. For example, genomes of thermophiles are generally smaller than those of mesophiles, with reduced number of some protein family members [14–16]. Horizontal gene transfer is thought to be important driving force for thermophilic adaptation. For instance, reverse gyrase, which was shown to have heat-protective DNA chaperone activity [17], is considered to be transferred from archaea to bacteria [18, 19].

Metabolism varies widely among thermophiles and general trends are hard to discern as it is extremely difficult to distinguish between the effect of speciation versus adjustment to extreme environments. For example, three main glycolytic pathways are used by bacteria in general: the traditional Embden-Meyerhof (EM) glycolysis, the Entner-Doudoroff pathway, and the pentose phosphate pathway; all three can also be found in different thermophilic bacteria [20–23]. In archaea, however, only modified variants of classical sugar degradation pathways were identified [23, 24]. For example, *Pyrococcus furiosus* contains a nontraditional variation of EM glycolysis, in which ADP-dependent kinases are involved and glyceraldehyde-3-phosphate is converted directly to 3-phosphoglycerate, with no thermolabile 1,3-biphosphoglycerate intermediate present as in traditional glycolysis [25, 26]. Even within the same taxonomic domain, sets of used pathways may vary. Flux analysis of three extremely thermophilic bacteria indicated that the metabolisms of the studied thermophiles were highly distinct, with differences in such pathways as amino acid or NADPH metabolism [27]. However, a communality is that all three strains relied heavily on glycolysis and the TCA cycle. Studies on *Thermus thermophilus,* possibly the most well-studied thermophile, revealed alternative pathways of amino acids synthesis, with lysine being synthesized by alpha-aminoadipate pathway instead of diaminopimelate pathway [28] and homocysteine produced from an alternative precursor, O-acetyl-L-homoserine [29]. *T. thermophilus* is also known to produce a variety of polyamines, the most common ones, spermidine and spermine, are synthesised using a distinct pathway from L-arginine via aminopropyl agmatine [30]. Analysis of complete thermophilic genomes is a widely used method of finding both novel metabolic pathway, as well as enzymes of potential biotechnological use [31–33]. An alternative pathway of menaquinone (MK) synthesis was discovered when no genes from the classical pathway were found in genomes of some MK-producing microorganism [34]. Genes involved in the novel MK synthesis pathway are present in a range of thermophilic microorganisms, which may play a role in adaptation to growth at high temperatures, as one of the intermediates in the classical pathway, isochorismate, is known to be thermolabile [35].

Several studies avoid the limitations associated with comparing a small number of thermophilic versus mesophilic species, thereby identifying more general trends, by performing metagenomic analysis of environmental samples from extreme habitats [36, 37]. A study comparing metagenomes obtained from cold and hot deserts found thermophiles having a higher number of genes involved in metabolism and transport of carbohydrates and secondary metabolites [38]. Similar results were found in other studies of metagenomes from hot springs in India, where abundance of KEGG [39, 40] pathways was investigated [41, 42].

In this work I investigate metabolic trends in adapting to thermophilicity by comparing the presence of metabolic enzymes over many thousands of organisms, growing at various temperatures. I identify 319 individual enzymatic reactions that are preferentially present at either low or high temperatures, indicating metabolic adaptations. I identify eight pathways which are over-represented in metabolic changes. These may represent parts of metabolism that are particularly important for the adaptation to growth at different temperatures. Finally, I show that 33 protein domains of unknown function (DUFs) likewise correlate with growth temperature, a result that may provide important clues to their function. Enabling this approach is a unique dataset of 21,498 organisms with their growth temperatures, which I obtained through data mining of publicly available organism culturing protocols from major culture collection centers. I make this data available to enable researchers to gain additional insights into evolutionary temperature-dependent adaptations.

## Results

### Culture collection center websites are a rich source for growth temperature data

**Fig. 1:**
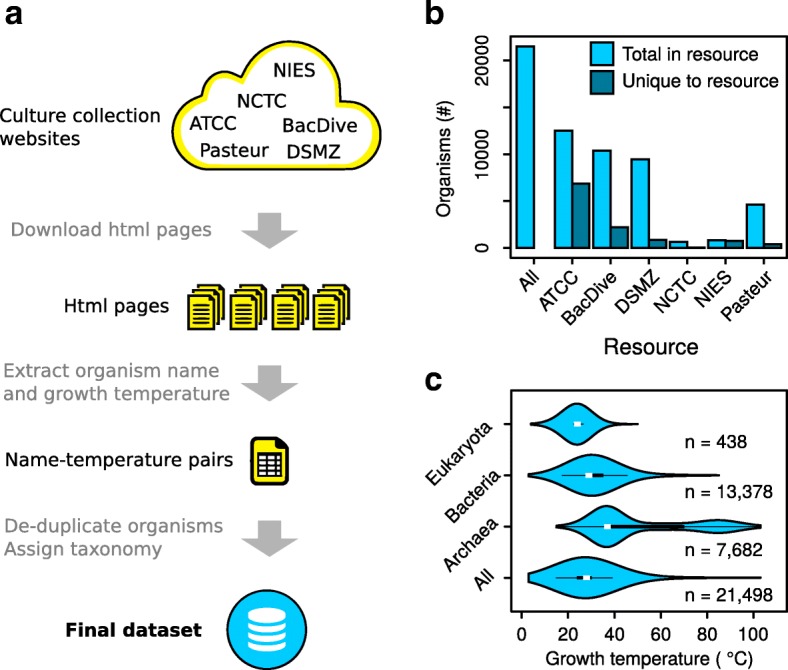
Culture collection centers represents a rich source for growth temperature data. **a** An overview schematic showing how the growth temperature dataset was obtained. **b** A database comparison showing which provided the largest number of unique organisms not present in the others. Total in resource shows the total number of organisms in each database, unique to resource shows how many organisms in that database were unique. **c** A violin-plot showing the growth distribution of growth temperatures in the dataset for each of the three domains of life. The white dot represents the median. The broad black bar represents the upper and lower quartile, which contain 50% of the data points. The thin black lines represent the upper and lower adjacent values. The outer plot shape is a kernel density plot that visualizes the probability distribution of the data

The hypothesis underlying this project is that, given a sufficiently large dataset with organism growth temperatures, meaningful correlations between growth temperatures and biological adaptations can be made. I reasoned that publicly available culturing protocols from microorganisms in major stock collection centers may form a resource that could be mined for such growth temperatures. Custom software in Python (http://www.python.org) was designed to systematically download all of the public web pages containing organism information from four stock collection centers: ATCC (http://www.lgcstandards-atcc.org), DSMZ (http://www.dsmz.de), NCTC (http://www.phe-culturecollections.org.uk) and NIES (http://www.shigen.nig.ac.jp). Each of these pages were then mined for the organism name and the growth temperature (Fig. 1a). A fifth stock center, the Institute Pasteur stock collection (http://www.research.pasteur.fr/en/team/biological-resources-center/) provided an excel table with organism names and growth temperatures upon request. Finally, organism growth temperatures from the BacDive database [1, 2] were collected using scripts interfacing with the public API. This procedure resulted in over 160,000 records, many of which were separate depositions for the same species. It is important to note that these records hold temperatures reported by a very large number of individual investigators for culturing organisms in a variety of growth conditions. In some cases they will represent true optimal growth temperatures, in others they may merely represent the temperatures at which the organisms were grown by that investigator, without the true optimum having been determined.

Organism names were collapsed to the species level (ignoring strain designations) and growth temperatures within the each species averaged. This procedure was performed to facilitate downstream taxonomic mapping and cross-database comparisons (see below). The approach is justified as the growth temperature of 99.7% of all strains in the dataset is less than 10 °C away from the calculated species mean and 96.4% is less than 5 °C away (Additional file 1: Figure S1). The number of organisms in each of the databases was compared (Fig. 1b). A total of 21,498 unique organism names combined with their growth temperature were obtained. ATCC and BacDive had the greatest number of unique organisms that were not present in any other database, with 6,852 and 2,211 respectively. In order, DSMZ, NCTC, NIES and Pasteur had 854, 44, 749 and 415 organisms that were uniquely present in each of the databases.

Each organism name in the dataset was mapped to a taxonomic identifier using the NBCI taxonomy resource (https://www.ncbi.nlm.nih.gov/taxonomy). The data from all databases were combined into one non-redundant dataset, which is referred to as the growth temperature dataset. The majority of organisms in the dataset (62%) is made up of bacteria (Fig. 1c). Most of these are psychrophiles and mesophiles. Only a small number are thermophiles. Archaea make up 36% of all organisms in the dataset, with a more even distribution between mesophiles, thermophiles and hyperthermophiles. Eukaryotes, mainly comprising fungi and protists, make up only 2% of the dataset and encompass both psychrophiles and mesophiles. In general the dataset contains many more psychrophiles and mesophiles than thermophiles and hyperthermophiles (Fig. 1c). The growth temperature dataset is made available for re-use by other researchers as a tab-delimited file as well as in the xml format at Zenodo (https://zenodo.org/; doi: 10.5281/zenodo.1175609).

### Growth temperatures correlate strongly with mean enzyme optima

**Fig. 2:**
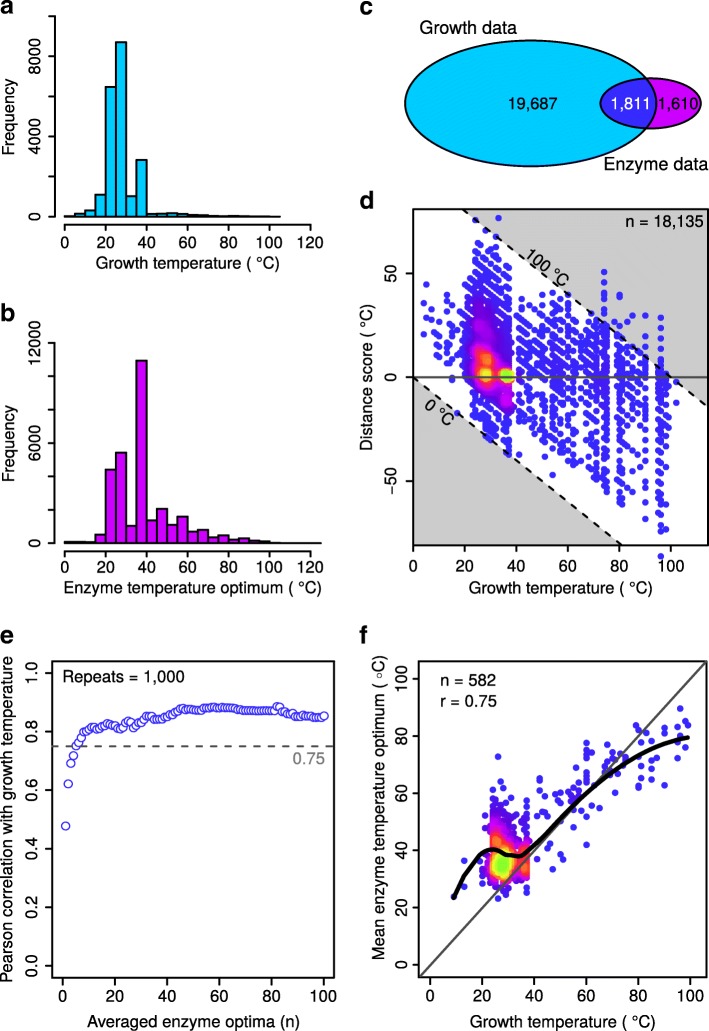
Growth temperatures correlate strongly with mean enzyme optima. **a** The distribution of growth temperatures for all organisms in the growth temperature dataset. **b** The distribution of experimentally determined optima for the 31,826 enzymes obtained from the BRENDA database. **c** A Venn diagram showing the number of organisms present in each of the growth temperature dataset, the enzyme temperature dataset, as well as the number of organisms present in both. **d** Distance score of individual enzyme temperature optima with growth temperature. The horizontal gray line indicates perfect agreement between the two values. The two diagonal dashed lines indicate absolute enzyme temperature optima of 0 °C and 100 °C. The colors indicate the spot density with brighter and more green colors indicating higher density. **e** A sensitivity plot showing the Pearson correlation coefficient between enzyme temperature optima and growth temperatures. Each data point represents the correlation coefficient obtained between the mean temperature optimum of n sampled enzymes in the range 1 < = n < = 100 and growth temperature. The average of five enzymes or more achieves a correlation coefficient above 0.75. **f** A scatterplot comparing the growth temperature of organisms with more than five reported enzyme temperature optima with the mean temperature optimum of all enzymes reported for that organism. The diagonal gray line indicates perfect positive correlation. The thick black line represents a locally weighted polynomial regression. The Pearson correlation coefficient is given

The growth temperature dataset was validated by investigating the correlation between the growth temperature of each organism and the temperature optima of enzymes from that organism. For this validation all experimentally determined enzyme temperature optima were extracted from the BRENDA enzyme database [43] (https://www.brenda-enzymes.org). The resulting data contained experimentally determined temperatures of maximal enzymatic activity (the “Temperature optimum” data category in BRENDA) for 31,826 enzymes, sourced from 3,421 organisms. This dataset is referred to as the enzyme temperature dataset. The overall distribution of growth temperatures (Fig. 2a) differ compared to the overall distribution of enzyme temperature optima (Fig. 2b). The majority (88%) of growth temperatures fall in the range between 20 °C and 40 °C, with an abrupt drop in the number of organisms grown at temperatures over 40 °C. In comparison, the majority of enzyme temperature optima also fall in the range between 20 °C and 40 °C (64%), but their distribution decreases much less drastically above this temperature range.

To be able to correlate growth temperatures with enzyme optima, organisms present in both datasets were identified (Fig. 2c). Of the 21,498 organisms with growth temperatures and the 3,421 organisms with enzyme temperature optima only 1,811 were present in both datasets. From these the experimentally determined temperature optima for 18,135 enzymes were available. A simple distance score was computed by subtracting each organisms growth temperature from each individual enzymes temperature optimum (Fig. 2d). Enzymes with optima exactly at the growth temperature have a distance score of 0 °C. Those which have an optimum higher than the growth temperature have a positive distance score, with a magnitude equal to the temperature difference. Enzymes with an optimum lower than the growth temperature have a negative distance score. Enzymes with optima between 20 and 100 °C could be found throughout the entire growth temperature range, indicating that not all enzymes match the organism growth temperatures (Fig. 2d). Despite these deviations from the expected, half (51%) of the enzyme measurements were in fact within ±10 °C of the growth temperature and 67% were within ± 15 °C of the growth temperature, showing that growth temperature may be a good indicator for the majority of enzyme optima.

This correlation was further investigated by comparing each organisms growth temperature with average enzyme optima calculated with an increasing number of averaged enzymes from that organism, ranging from 1 to 100 (Fig. 2e). There is a clear trend for stronger correlation between growth and enzyme temperatures when increasing the number of averaged enzymes. This result shows that a single randomly chosen enzyme (*n* = 1) is an imprecise predictor of growth temperature with a Pearson correlation of 0.48. (Fig. 2e, bottommost circle). However, the mean optimum of at least five enzymes does display a Pearson correlation coefficient greater than 0.75. It is expected that the optimal catalytic temperature of enzymes follows growth temperature, the high correlation seen between these two variables is therefore a validation of the growth temperature dataset.

### Many enzyme functions correlate with temperature

A range of biological questions might be investigated by correlating biological properties with the collected organism growth temperatures. These could include genomic, transcriptomic, proteomic, metabolomic, phenotypic or taxonomic properties. To test this idea, I correlated enzyme functions, classified by Enzyme Commission numbers (EC numbers), with growth temperatures in an effort to identify temperature-dependent metabolic adaptations. A limitation in this approach is that it only focuses on known enzyme functions and novel ones cannot be directly identified. Missing annotations as well as mis-annotations also introduce noise in this analysis. Furthermore, it is important to consider that the presence of an enzyme coding sequence in a dataset does not mean it is expressed and functional in a given organism.

To obtain a set of enzyme functions for analysis all 88. 6 million protein records from the UniProt database [44] (https://www.uniprot.org/) were downloaded. Of these, 43% (38.3 million) could be annotated with the growth temperature of the organism from which they came - using the growth temperature dataset. EC numbers, were subsequently obtained for each of the matched records, where present, using the UniProt ID mapping tool (https://www.uniprot.org/uploadlists/). This resulted in the mapping of 3,551 unique EC numbers, which is approximately half of those currently listed in the BRENDA database. For the subsequent analysis Eukaryotes were excluded due to their limited range of growth temperatures in the dataset.

**Fig. 3:**
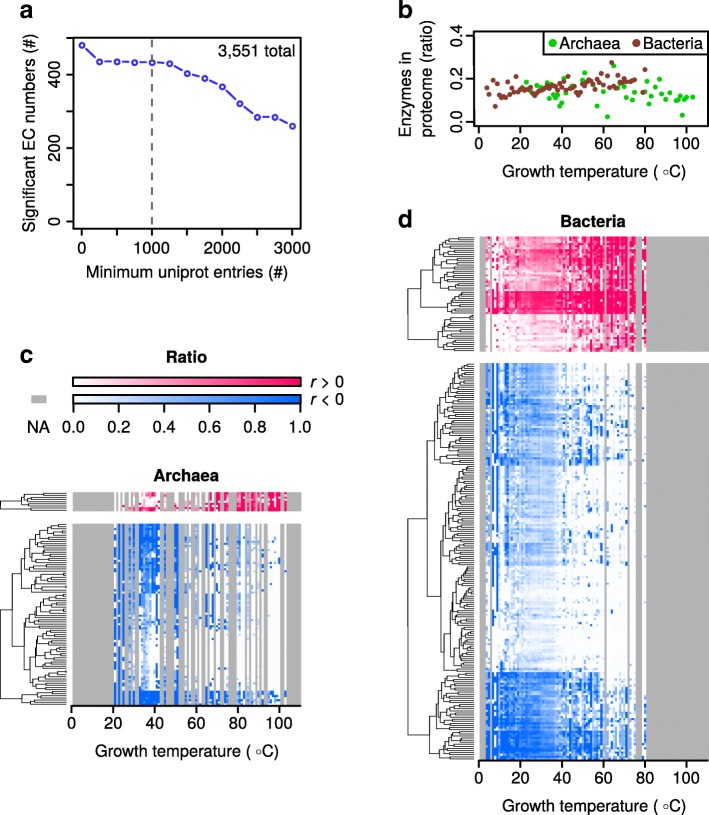
Many enzyme functions correlate with temperature. **a** A sensitivity plot showing the number of significant EC numbers (corrected *p*-value < 0.01) obtained at different cutoffs for minimum number of UniProt entries per organism. The gray dashed line indicates the selected cutoff. **b** Enzyme annotations as a proportion of the proteome. Each point represents the mean for all organisms growing at a given temperature. The means for archaea and bacteria were calculated separately. **c** Heat-maps showing how the occurrence of significant EC numbers change with growth temperatures in archaea. Each row represents a different EC number and each column a different growth temperature. All included EC numbers are statistically correlated (corrected p-value < 0.01) either positively (red) or negatively (blue) with growth temperature and a minimum phylogenetic distance score of six (see Methods for details). For each reported growth temperature, the ratio of organisms containing each specific EC annotation is shown by the intensity of the color. Gray indicates temperatures for which no growth temperatures are available in the dataset. **d** Analysis and color scale as in c, but performed with data from bacteria. *r* indicates Spearman’s correlation coefficient

The growth temperature dataset is highly skewed toward organisms growing between 20 °C and 40 °C (Fig. 1c). This skewing of the data would interfere with a correlation analysis. Therefore, for each individual EC number, the ratio of organisms having at least one protein carrying that annotation - versus those organisms that do not - was calculated to obtain a single value at each growth temperature. This results in a distribution of ratios over the analyzed temperatures for each individual EC number. A cutoff was set, including in the calculations only organisms with at least 1,000 protein records, as determined by a sensitivity plot (Fig. 3a). This cutoff was used to remove noise caused by organisms with very few entries in UniProt. The proportion of proteins annotated as enzymes remained approximately constant across growth temperatures in both archaea and bacteria (Fig. 3b).

In the growth temperature dataset there are proportionally more bacteria at low growth temperatures and proportionally more archaea at high growth temperatures (Additional file 2: Figure S2). Correlating EC numbers and growth temperatures for the entire dataset may therefore highlight those that differ between these two domains of life, instead of those representing a true signal for temperature adaptation. Bacteria and archaea were therefore analyzed separately. The Spearman correlation coefficient between the growth temperature and the enzyme occurrence ratios was calculated for each EC number and corrected *p*-values obtained (Additional file 3: Figure S3). A concern in this analysis is that it may result in false positives representing enzyme functions only present in closely related organisms with a limited growth temperature distribution. To remove such false positives - and retain only those that may represent a general strategy of temperature adaptation - a filtering step based on phylogenetic distance was performed. Enzyme functions with significant correlation (corrected p-value below 0.01) were thus filtered to retain only those with a wide phylogenetic distribution, indicated by a phylogenetic distance score of at least six (see Methods for details).

From the 3,551 mapped EC numbers a total of 319 unique EC numbers remaining after filtering (340 total instances when accounting for recurring EC numbers), nine show statistically significant positive correlation in archaea and 55 in bacteria (Fig. 3c and d, top panels; Additional file 5: significant_ec.tsv). Conversely, 86 enzymes from archaea show negative correlation with temperature and 190 in bacteria (Fig. 3c and d, bottom panel; Additional file 5: significant_ec.tsv). 14 EC numbers recur in both the archaeal and bacterial dataset with the same correlation and 7 EC numbers show opposite correlation in archaea compared to bacteria. Together, these 319 EC numbers may shed light on metabolic adaptations important for growth at different temperatures.

### Certain metabolic pathways are enriched for the correlated enzyme functions

To take the analysis one step further I investigated whether any part of metabolism was enriched for the enzyme functions identified in the previous step. Such enrichment would implicate specific metabolic pathways, or sets of related pathways, in the process of adapting to differing growth temperatures. The enzyme functions were mapped onto KEGG (http://www.genome.jp/kegg) pathways and a hypergeometric test was applied to test for enrichment. Eight out of 155 KEGG pathways were statistically enriched for the enzyme functions. These were: TCA cycle (KEGG pathway map00020), ubiquinone and other terpenoid-quinone biosynthesis (KEGG pathway map00130), purine metabolism (KEGG pathway map00230), cysteine and methionine metabolism (KEGG pathway map00270), pyruvate metabolism (KEGG pathway map00620), one carbon pool by folate (KEGG pathway map00670), methane metabolism (KEGG pathway map00680), and carbon fixation pathways in prokaryotes (KEGG pathway map00720).

**Fig. 4:**
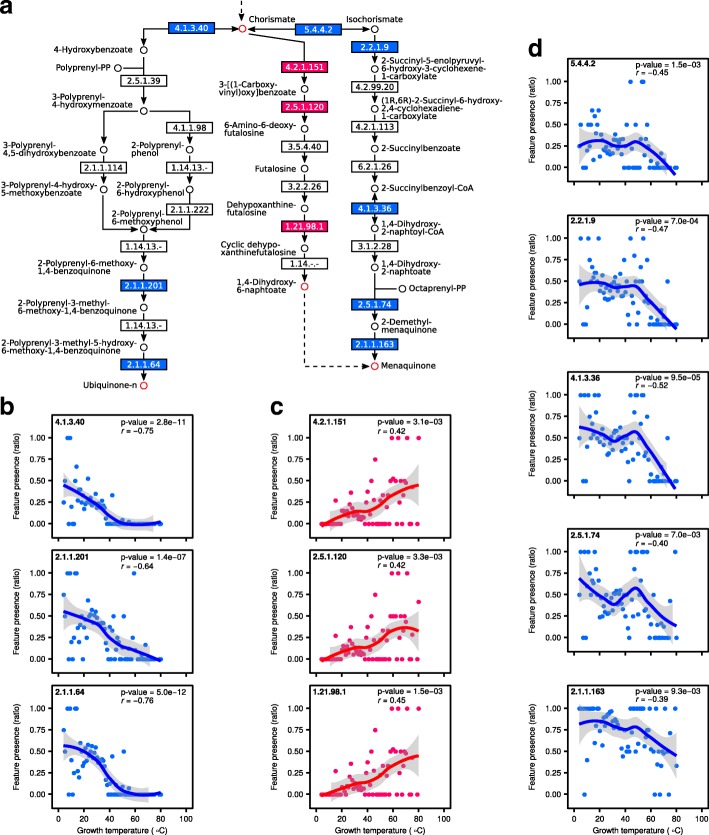
Certain metabolic pathways are enriched for the correlated enzyme functions. **a** A pathway diagram showing part of the KEGG pathway “ubiquinone and other terpenoid-quinone biosynthesis pathway” (map00130). Enzymes whose occurrence are significantly correlated with growth temperature (corrected p-value < 0.01) are shown in red (positive correlation) or blue (negative correlation). **b** The presence of enzymes participating in the biosynthesis of Ubiquinone changes with temperature. Each point indicates the occurrence of the EC number as a ratio of its presence in all organisms growing at a specific temperature. The line represents locally weighted polynomial regression with a 95% confidence band for the regression line indicated in gray. *r* indicates Spearman’s correlation coefficient. **c** The presence of enzymes participating in the biosynthesis of menaquinone via the futalosine pathway changes with temperature. **d** The presence of enzymes participating in the biosynthesis of menaquinone via the classical pathway changes with temperature

To highlight one of these pathways a portion of the “ubiquinone and other terpenoid-quinone biosynthesis pathway” is presented in Fig. 4a with data from bacteria. The ubiquinone synthesis pathway contains three enzymes that show negative correlation with growth temperature (Fig. 4b). Two different metabolic pathways lead to synthesis of menaquinone from chorismate. The first, so-called futalosine pathway, contains three enzymes that show positive correlation with temperature (Fig. 4c). The second pathway of menaquinone biosynthesis contains five enzymes that show negative correlation with growth temperature and are not present at high temperatures (Fig. 4d). These data suggest that an evolutionary adaptation to growth at high temperatures in many bacteria is to biosynthesize menaquinone via the futalosine pathway.

### Domains of unknown function correlate with temperature

**Fig. 5:**
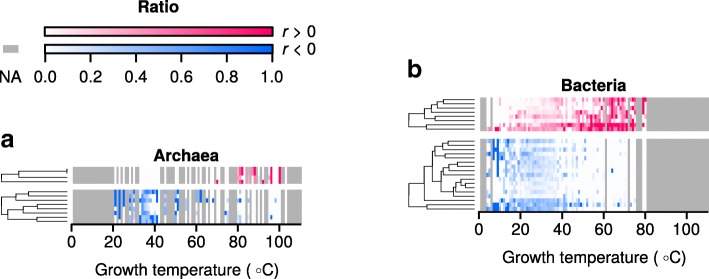
Domains of unknown function correlate with temperature. **a** Heat-maps showing how the occurrence of significant DUFs change with growth temperatures in archaea. Each row represents a different DUF and each column a different growth temperature. All included DUFs are statistically correlated (corrected p-value < 0.01) either positively (red) or negatively (blue) with growth temperature and a minimum phylogenetic distribution score of six (see Methods for details). For each reported growth temperature, the ratio of organisms containing each specific DUF annotation is shown by the intensity of the color. Gray indicates temperatures for which no growth temperatures are available in the dataset. **b** Analysis and color scale as in a, but performed with data from bacteria

The EC correlation analysis is limited in that it can only leverage enzyme functions that have been characterized. Unknown activities are “hidden” and cannot be correlated. To address this limitation I performed a final analysis where domains of unknown function (DUFs) were correlated with temperature in the same manner as the EC numbers. DUFs are domains that show a conserved pattern in protein primary sequences, but for which the function of proteins in which they occur is not known. These are advantageous for the analysis insofar as they can be identified from sequence data alone and one is therefore not limited to what is known through experimentation. Out of 3,918 total DUFs, 98 were were significantly correlated with temperature (Additional file 4: Figure S4), 33 of which also had a wide phylogenetic distribution (Fig. 5, Additional file 6: significant_duf.tsv). Four of the 33 were positively correlated in archaea, and eight were negatively correlated (Fig. 5a). Seventeen were positively correlated in bacteria and eight were negatively correlated (Fig. 5b). Two of these, DUF438 (PF04282) and DUF1957 (PF09210), were positively correlated in both archaea and bacteria. Also for negative correlation two domains, DUF3458_C (PF17432) and DUF1524 (PF07510), appeared in both archaea and bacteria.

## Discussion

The dataset collected in this work, comprising 21,498 organism names and growth temperatures, is considerably larger than those available through BacDive [1, 2], MediaDB [4] and IJSEM [6]. The comprehensiveness of the dataset is demonstrated by the fact that 43% of all entries in UniProt could be annotated with the growth temperature of the organism from which they came. This is particularly impressive considering that the dataset focuses on microbes and many organisms in the UniProt database do not fall in this category – for example mammalian or plant sequences. In the dataset there is a very high proportion of mesophilic organisms compared to thermophilic ones (Fig. 1c). The sharp drop in growth temperatures above 40 °C closely matches that seen by Barberan and colleagues [6]. To what extent this reflects a true distribution of growth temperatures of naturally occurring organisms, and to what extent this is a bias introduced by what researchers have chosen to study is unclear.

When comparing enzyme optima (the temperature with maximal catalytic activity as reported in the BRENDA database) with growth temperature there are two important aspects: First, proteins from mesophiles tend to be more stable than expected based on the growth temperature. Second, proteins from thermophiles and hyperthermiphiles tend to be less stable than expected (Fig. 2d and f). Together, the unexpectedly high stability of many enzymes coming from mesophilic organisms, and the unexpectedly low stability of many of the enzymes from thermophilic and hyperthermophilic organisms likely explains the smoothing above 40 °C of the enzyme temperature histogram (Fig. 2b), as compared to the growth temperature histogram (Fig. 2a).

The trend for mesophilic proteins to be catalytically active at higher temperatures than expected matches the observation made by Dehouck and colleagues [45]. A weak correlation between individual enzyme optima and growth temperature was shown in that study, albeit with a smaller dataset [45]. A novel insight gained in the current work is that the average catalytic optimum of at least five enzymes correlates strongly (Pearson correlation > 0.75) with growth temperature throughout the organisms analyzed (Fig. 2e and f). This provides an important validation of the accuracy of the growth temperature dataset.

It is surprising that many individual enzymes in thermophiles and hyperthermophiles have catalytic optima much lower than the growth temperatures (Fig. 2d). Indeed, even the average stability of all enzymes characterized from these organisms is on the order of 10 to 20 °C lower than the growth temperature (Fig. 2f). It has been proposed previously that temperature adaptation in thermophiles and hyperthermophiles may result from extrinsic factors for stabilizing the proteome – in addition to adaptations in protein sequence and fold. These may include compatible solutes [46] such as diglycerol phosphate [47], di-myo-inositol-phosphate [48], increased action by chaperones, higher protein turnover rates, molecular crowding, or other, as yet undiscovered mechanisms. The difference in growth temperatures and enzyme optima in thermophiles presented here supports the idea that such extrinsic factors are important.

The need to evolve adaptations to globally stabilize the proteome likely arises from the fact that a random mutation in a thermostable protein is more likely to lead to decreased stability compared to a random mutation in a moderately stable protein [49]. This effect becomes more pronounced with increasing growth temperature and makes it increasingly difficult to evolve more stable proteins. This also makes thermophiles more sensitive to random mutations than mesophiles and essentially puts a “speed limit on evolution” [49]. I speculate that evolving adaptations (extrinsic factors) that stabilize the entire proteome provides a solution to both the difficulty of evolving highly stable proteins as well as a way to alleviate some of the risk posed by random mutations, allowing the organisms to tolerate higher mutation rates. Nature provides ample examples of other evolutionary adaptations that modify the intracellular physiochemical environment to ensure the function of proteins that function poorly in ambient conditions. For example, developing heterocysts to ensure microoxic environments for N_2_ fixation by nitrogenase in filamentous cyanobacteria [50], and Krantz anatomy to concentrate CO_2_ for carbon fixation by Rubisco in C4 plants [51].

For both archaea and bacteria there are several times more enzymes whose occurance are negatively correlated with temperature (more present at low growth temperatures) than there are positively correlated ones (Fig. 3c and d; Additional file 5: significant_ec.tsv). Specifically, there were 64 enzyme functions that were positively correlated with temperature and 276 that were negatively correlated. I speculate that this difference is a direct result of mesophiles having been studied to a higher degree than thermophiles and hyperthermophiles, resulting in a greater number of known enzyme functions important for growth at mesophilic temperatures. In a logical extension of this argument I propose that a large number of enzyme functions likely remain to be discovered in thermophilic organisms. Each of the 319 enzymes here shown to be differentially present at various growth temperatures highlight important targets for future hypothesis generation and experimental investigation. In particular, further study is needed to determine whether the correlation reflects true causality.

Of the eight KEGG pathways - with significant over-representation of enzymes changing with temperature - four were previously identified as over-represented in metagenomic studies of high temperature habitats [41, 42]: carbon fixation pathway in prokaryotes, pyruvate metabolism, methane metabolism and purine metabolism. The TCA cycle, another enriched KEGG pathway, is known to be extensively utilized in three thermophilic bacteria [27]. In my approach, by identifying pathways with enzymes whose occurrence strongly correlates with growth temperatures, I strengthen the argument that these pathways are under evolutionary pressure in temperature adaptation. It therefore represents a clear advancement over comparing small numbers of mesophilic and thermophilic organisms and highlights the parts of metabolism under higher evolutionary pressure to change with temperature.

One of the enriched pathways identified here contains enzymes of quinone metabolism. A minority of bacteria synthesise ubiquinone [52, 53]. The results obtained in this study show that among these that do synthesise it, the occurrence of ubiquinone biosynthesis genes decrease with temperature (Fig. 4a and b). Menaquinone synthesis occurs via two pathways, classical and futalosine, and genes involved in the futalosine pathway are present in a range of thermophilic microorganisms [34]. Here, I provide evidence that the futalosine pathway is not only present in thermophilic organisms, but is in fact the prevailing one at high growth temperatures in bacteria. I speculate that this may be an evolutionary adaptation reflecting the fact that the classical pathway contains the thermolabile intermediate isochorismate [35].

To expand my analysis to protein functions that have not been determined I correlated the occurrence of domains of unknown function (DUFs) with temperature. The 33 significant domains identified (Fig. 5, Additional file 6: significant_duf.tsv) may represent functions important to adaptation to growth at diverse temperatures. Using computational approaches to gain insights regarding the function of DUFs has been done previously. For example, a list of 238 essential DUFs (eDUFS), were identified based on their presence in essential proteins in bacteria [54]. Another approach made use of remote similarity detection to establish structure-function relationships for 614 DUF families, thus providing clues to their function [55]. Experimental approaches, notably that of structural genomics, has also been employed to gain additional information on DUFs [56]. To my knowledge the approach outlined here is the first to provide evidence connecting DUFs with putative temperature adaptations in bacteria and archaea. As such, these 33 domains provide important starting points for future studies.

## Conclusions

In this study I mine and make available growth temperatures for 21,498 organisms. This dataset is comprehensive and allows the growth temperature annotation of 43% of all sequences in UniProt. These annotations can be leveraged for correlating enzyme functions and domain-level annotations to highlight growth-temperature dependent changes in their occurrence. In a few cases this analysis confirmed what is known, such as the preference for menaquinone biosynthesis through the futalosine pathway in bacteria growing at high temperatures. In other cases the results represent important starting points for future studies, for example in highlighting DUFs whose occurrence change with temperature. I believe that the dataset will be a valuable community resource and will find additional, important, uses in correlating genomic, transcriptomic, proteomic, metabolomic, phenotypic or taxonomic properties with temperature in future studies.

## Methods

### Obtaining the growth temperature dataset

Organism growth condition data was downloaded from the four culture collection centers ATCC, DSMZ, NCTC and NIES in the form of html files. For each organism record the scientific name and growth temperature was extracted using custom scripts in Python version 2.7, which are freely available for re-use (10.5281/zenodo.1458278). Data relating to organism names and growth temperatures from the Pasteur institute was obtained as an Excel file (personal communication). Data from the BacDive database was obtained using custom scripts in Python by interfacing with the public application programming interface (API). Records from ATCC, DSMZ and BacDive reflect those available in July of 2017. Records from Pasteur, NCTC and NIES reflect those available in the first months of 2015. Subspecies names or strain designations were removed from all organisms and the reported growth temperatures for records with the same species name were averaged and rounded to the closest integer. Organism names were further matched to taxonomic identifiers (TaxId) using the NCBI taxonomic database from July 2017; organisms for which none could be obtained were removed from the dataset. The taxonomic lineage for each organism was subsequently obtained by querying the NCBI database with the TaxIds and using the eutils resource (https://www.ncbi.nlm.nih.gov/). All growth temperatures were validated to ensure that they fall in the range of − 5 to 130 °C.

### Obtaining the enzyme temperature dataset

All available experimentally determined enzyme temperature optima were extracted from the BRENDA enzyme database release 2017.2 (July 2017) with Python scripts using the Zolera SOAP package (https://pypi.python.org/pypi/ZSI/) interacting with the public BRENDA API. To de-duplicate data coming from the same enzyme the temperature optima from enzymes with the same EC number were averaged within each organism and rounded to the closest integer. TaxId was obtained for each organism using the NCBI eutils resource. Organisms for which no TaxId could be found were removed from the dataset.

### Comparing the growth- and enzyme temperature datasets

For each organism the collected growth temperatures were compared with enzyme temperature optima. The organisms present in both datasets were identified through matching of species names.

For Fig. 2d a simple distance score was computed by subtracting each organisms growth temperature from each individual enzymes temperature optimum. This was done in order to more easily visualize the magnitude of the difference between the two variables. Positive distance scores represent enzymes that have catalytic optima higher than the growth temperature. Negative scores represent enzymes that have optima lower than the organism growth temperature. This procedure was used instead of z-normalization as the goal of this plot was to visualize the actual, biologically relevant, temperature difference between the two variables and not just a normalized magnitude difference.

For Fig. 2e the goal was to evaluate how well the arithmetic mean of measured enzyme temperature optima for a specific organism correlates with the organisms growth temperature. Specifically, the analysis focuses on showing how the strength of that correlation changes with the number of individual enzyme optima used to calculate the mean. The following steps were performed: For each of the 1,811 organisms present in both the growth temperature and enzyme temperature datasets n datapoints for enzyme optima (where n represents each integer in the range 1 to 100) were sampled at random without replacement and the mean temperature calculated. In each iteration only organisms having equal to or more than n reported enzyme optima were included in the calculations. The Pearson correlation between these mean enzyme temperature optima and the organism growth temperatures were subsequently calculated. The calculation was repeated 1,000 times for each n and the mean of these calculations is reported.

The mean enzyme temperature optima used in Fig. 2f were calculated through simple averaging of all reported enzyme optima from each organism. Based on the results observed in Fig. 2e, organisms for which fewer than five enzyme optima were reported were excluded. A locally weighted polynomial regression (LOESS) was calculated using the loess() function (authored by B. D. Ripley, based on the cloess package of Cleveland, Grosse and Shyu.) [57] from the “stats” base package in R version 3.4.3.

### Obtaining and filtering UniProt data

SwissProt and TrEMBL protein sequences were downloaded as fasta files from UniProt release 2017_07. A series of data matching and filtering steps were performed, as outlined below, to obtain a set of EC numbers and Pfam domains to analyze. The fasta files from both resources were parsed to extract the species name belonging to each UniProt sequence identifier. Where possible, each of these species were assigned a growth temperature through name matching with the growth temperature dataset. UniProt identifiers belonging to organisms with no assigned growth temperature were removed from further analysis. Additionally, identifiers belonging to organisms with fewer than 1,000 sequences in the resource were discarded (Fig. 3a). UniProt identifiers remaining after this initial filtering, EC annotations and Pfam domains, where available, were obtained using the UniProt ID mapping tool (https://www.uniprot.org/uploadlists/). To create the EC dataset UniProt identifiers not annotated with an EC number and those with incomplete EC numbers (for example 1.14.-.-) were discarded. To create the Pfam domain dataset UniProt identifiers not annotated with a Pfam domain were discarded. The remaining UniProt identifiers, all annotated with a species name, growth temperature, and either an EC number or Pfam domain were each subjected to correlation analysis with growth temperature.

### EC correlation analysis

The filtered UniProt EC dataset described above is skewed, disproportionately containing identifiers annotated with growth temperatures in the range 20 to 40 °C. To remove this skewing the ratio between the number of organisms carrying a protein with a specific EC annotation versus the number of organisms that do not was calculated - separately at each growth temperature. This processing step results in a dataset where the occurrence of each EC number is represented by a single ratio, between 0.0 and 1.0, at each growth temperature. The Spearman correlation between this ratio and the growth temperature was subsequently calculated for each EC number. In this calculation, identifiers from bacteria and archaea were treated separately. This separation was done since the growth temperature distribution of archaea and bacteria differ, with more archaea growing high temperatures and more bacteria growing at low temperatures. Analyzing the identifiers from these domains together may thus erroniously result in the identification of EC numbers that differ between bacteria and archaea. The resulting *p*-values were corrected for multiple testing with the p.adjust() function from the “stats” base package in R version 3.4.3, using false detection rate (FDR) according to Benjamini and Hochberg [58]. This analysis ultimately generated a set of EC numbers either significantly positively or negatively correlated with temperature.

In a final step of the analysis, EC numbers with a narrow phylogenetic distribution were removed. Organism relatedness was calculated from the KEGG taxonomy (http://www.genome.jp/kegg/genome.html). Organisms were treated as leaves in a rooted tree. A phylogenetic distance score was used that represents the number of nodes that two leaves (organisms) are separated by. The precise calculation will be published in a forthcoming paper describing the GECKO toolbox. For the work described here, a MatLab matrix containing the distance scores was downloaded from a GitHub repository (https://github.com/SysBioChalmers/GECKO/tree/master/databases/PhylDist.mat) and exported to a text file. The distance is zero for two organisms that share the same taxonomic levels but just differ on the organism name level. The maximum score in this scheme is eight, indicating two organisms from different domains of life. EC numbers whose occurance significantly correlate with temperature were filtered to retain those present in at least two organisms with a distance of six or more.

### Domain correlation analysis

The filtered UniProt Pfam domain dataset described above was filtered to retain only those corresponding to DUFs. The ratio between the number of organisms carrying a protein with a specific domain annotation versus the number of organisms that do not was calculated as described above. Calculation of the correlation of these ratios with temperature, *p*-value correction, and filtering for phylogenetic diversity was likewise performed as described above.

### Identifying metabolic pathways with significantly over-represented temperature-correlated enzyme functions

To identify metabolic pathways, or collection of such pathways, with statistically significant over-representation of temperature-correlated EC numbers the following steps were undertaken: All available KEGG pathway data was downloaded as xml files using the KEGG representational state transfer (REST) API (http://www.kegg.jp/kegg/rest/keggapi.html). The EC numbers listed in each of these pathways were extracted using a Python script. The occurrence of EC numbers in each of these pathways were matched against the EC numbers that significantly correlate with temperature to find the overlap. Finally, a Fischer hypergeometric test [59] was performed, using the phyper() function from the “stats” base package in R version 3.4.3, to test for enrichment of temperature-correlated EC numbers in these pathways. The resulting p-values were adjusted for multiple testing using FDR, as outlined above, and those pathways with a p-value smaller than 0.05 reported.

## Additional files

Additional file 1: **Figure S1.** The effect of averaging growth temperatures from different strains of the same species is small. Each of the over 160,000 individual records obtained from the culture collection centers were analyzed to see to what extent the reported strain growth temperature differs from that of the calculated average of all strains of a species. (PDF 9 kb)

Additional file 2: **Figure S2.** The abundance of archaeal species compared to bacterial species changes with temperature. Each point indicates the ratio of bacterial and archaeal species as a proportion of the total number of species for each of the growth temperatures in the dataset. (PDF 39 kb)

Additional file 3: **Figure S3.** EC numbers both negatively and positively correlated with growth temperature can be identified. **a** The correlation between the occurrence of unique EC number annotations in species and their growth temperature is shown in archaea. Each point indicates the Spearman correlation coefficient and the corrected p-value (adjusted by false discovery rate) for a single EC number. Significant EC numbers (corrected p-value < 0.01) with positive correlation are colored red, those with negative correlation are colored blue. **b** The correlation between the occurrence of unique EC number annotations in species and their growth temperature is shown in bacteria. Analysis and color scale as in A. (PDF 832 kb)

Additional file 4: **Figure S4.** Domains of unknown function (DUFs) both negatively and positively correlated with growth temperature can be identified. **a** The correlation between the occurrence of unique DUFs in species and their growth temperature is shown in archaea. Each point indicates the Spearman correlation coefficient and the corrected p-value (adjusted by false discovery rate) for a single DUF. Significant DUFs (corrected p-value < 0.01) with positive correlation are colored red, those with negative correlation are colored blue. **b** The correlation between the occurrence of unique DUFs in species and their growth temperature is shown in bacteria. Analysis and color scale as in A. (PDF 166 kb)

Additional file 5: **significant_ec.tsv.** A tab-delimited data file containing a total of 319 unique EC numbers that are significantly correlated with temperature in archaea or bacteria. Respectively, the data headers “domain” and “ec” indicate the domain of life and the EC number. The table indicates the Spearman correlation coefficient (“spearmah_rho”) and the corrected p-value (adjusted by false discovery rate, “pval_norm”). Only EC numbers with a p-value less than or equal to 0.01 are included. (TSV 11 kb)

Additional file 6: **significant_duf.tsv.** A tab-delimited data file containing a total of 33 unique DUFs that are significantly correlated with temperature in archaea or bacteria. Respectively, the data headers “domain”, “pfam_id” and “duf_id” indicate the domain of life, the Pfam identifier and the DUF identifier. The table indicates the Spearman correlation coefficient (“spearmah_rho”) and the corrected p-value (adjusted by false discovery rate, “pval_norm”). Only DUFs with a p-value less than or equal to 0.01 are included. (TSV 1 kb)

## Abbreviations

API: Application programming interface
DUF: Domain of unknown function
EC: Enzyme commission
FDR: False Detection Rate
LOESS: Locally weighted polynomial regression
MK: Menaquinone
REST: Representational State Transfer
