# Supplementary figures and images for: Correlating enzyme annotations with a large set of microbial growth temperatures reveals metabolic adaptations to growth at diverse temperatures
Source: BMC Microbiol. 2018 Nov 6;18:177. doi: 10.1186/s12866-018-1320-7 (PMC6219164; doi:10.1186/s12866-018-1320-7)

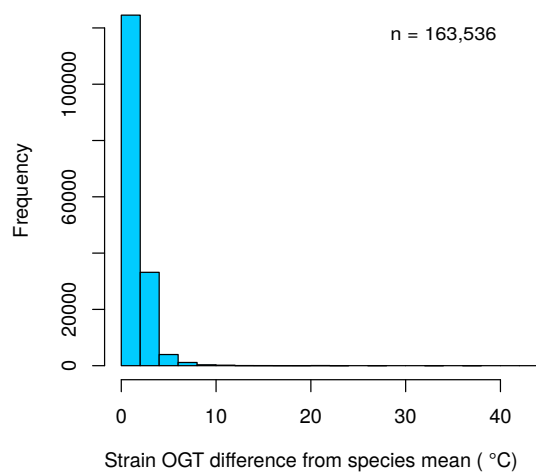

Supplement: Supplementary file 1 — Figure S1. The effect of averaging growth temperatures from different strains of the same species is small. Each of the over 160,000 individual records obtained from the culture collection centers were analyzed to see to what extent the reported strain growth temperature differs from that of the calculated average of all strains of a species. (PDF 9 kb) [file 12866_2018_1320_MOESM1_ESM.pdf]

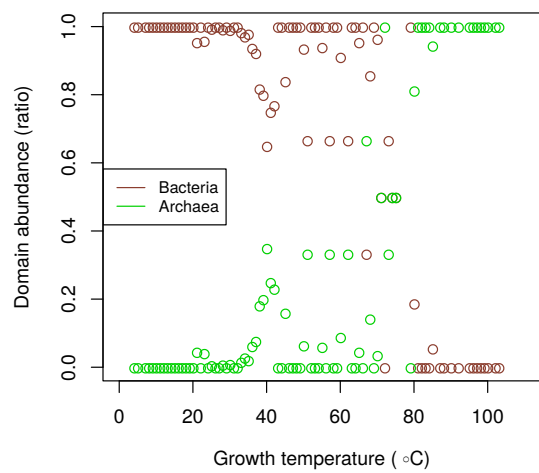

Supplement: Supplementary file 2 — Figure S2. The abundance of archaeal species compared to bacterial species changes with temperature. Each point indicates the ratio of bacterial and archaeal species as a proportion of the total number of species for each of the growth temperatures in the dataset. (PDF 39 kb) [file 12866_2018_1320_MOESM2_ESM.pdf]

a

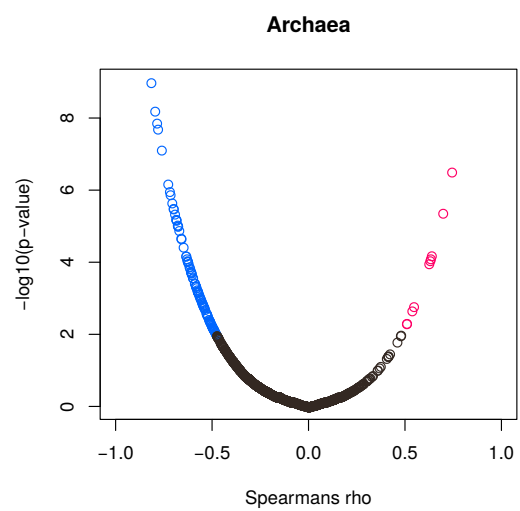

b

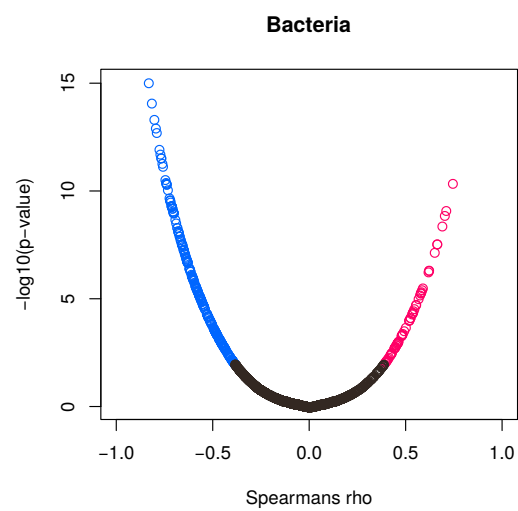

Supplement: Supplementary file 3 — Figure S3. EC numbers both negatively and positively correlated with growth temperature can be identified. a The correlation between the occurrence of unique EC number annotations in species and their growth temperature is shown in archaea. Each point indicates the Spearman correlation coefficient and the corrected p-value (adjusted by false discovery rate) for a single EC number. Significant EC numbers (corrected p-value < 0.01) with positive correlation are colored red, those with negative correlation are colored blue. b The correlation between the occurrence of unique EC number annotations in species and their growth temperature is shown in bacteria. Analysis and color scale as in A. (PDF 832 kb) [file 12866_2018_1320_MOESM3_ESM.pdf]

a

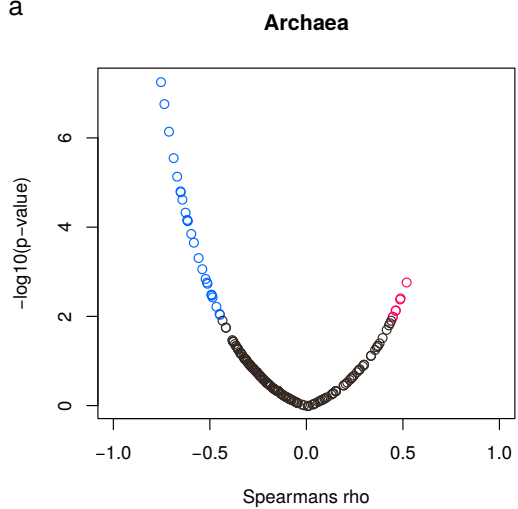

b

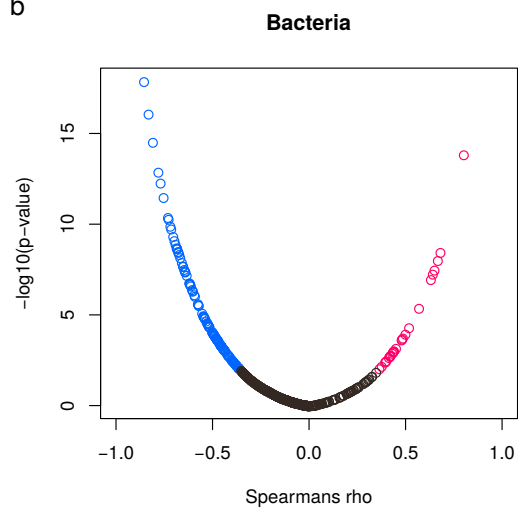

Supplement: Supplementary file 4 — Figure S4. Domains of unknown function (DUFs) both negatively and positively correlated with growth temperature can be identified. a The correlation between the occurrence of unique DUFs in species and their growth temperature is shown in archaea. Each point indicates the Spearman correlation coefficient and the corrected p-value (adjusted by false discovery rate) for a single DUF. Significant DUFs (corrected p-value < 0.01) with positive correlation are colored red, those with negative correlation are colored blue. b The correlation between the occurrence of unique DUFs in species and their growth temperature is shown in bacteria. Analysis and color scale as in A. (PDF 166 kb) [file 12866_2018_1320_MOESM4_ESM.pdf]
